# Annual report of National Clinical Database-Breast Cancer Registry in 2021: characteristics categorized by body mass index and menopause status

**DOI:** 10.1007/s12282-025-01698-3

**Published:** 2025-04-21

**Authors:** Takaaki Konishi, Hiraku Kumamaru, Naoki Niikura, Yasuaki Sagara, Minoru Miyashita, Takayuki Iwamoto, Naoko Sanuki, Kenta Tanakura, Masayuki Nagahashi, Masayuki Yoshida, Masahiro Kawashima, Takayuki Kinoshita, Shinsuke Sasada, Naoko Kinukawa, Shigehira Saji, Takanori Ishida, Naruto Taira

**Affiliations:** 1 Department of Breast and Endocrine Surgery, Graduate School of Medicine, The University of Tokyo, Tokyo, Japan; 2 Department of Healthcare Quality Assessment, University of Tokyo, 7‑3‑1 Hongo, Bunkyo‑Ku, Tokyo 113‑8655 Japan; 3 Department of Breast Oncology, Tokai University School of Medicine, 143 Shimokasuya, Isehara, Kanagawa 259‑1193 Japan; 4 Department of Breast Surgical Oncology, Social Medical Corporation Hakuaikai, Sagara Hospital, 3-28 Matsubara, Kagoshima, 892-833 Japan; 5 Department of Breast and Endocrine Surgical Oncology, Tohoku University Graduate School of Medicine, Seiryo‑Machi, Aoba‑Ku, Sendai, 980‑8574 Japan; 6 Department of Breast and Thyroid Surgery, Kawasaki Medical School Hospital, Kurashiki, Japan; 7 Department of Radiology, Keio University School of Medicine, 35 Shinanomachi, Shinjuku-ku, Tokyo 160-8582 Japan; 8 Plastic and Reconstructive Surgery, Mitsui Memorial Hospital, 1 Kanda‑Izumicho, Chiyoda‑Ku, Tokyo 101‑8643 Japan; 9 Department of Surgery, Division of Breast and Endocrine Surgery, School of Medicine, Hyogo Medical University, 1‑1 Mukogawa, Nishinomiya, Hyogo 663‑8501 Japan; 10 Department of Diagnostic Pathology, National Cancer Center Hospital, 5‑1‑1 Tsukiji, Chuo‑Ku, Tokyo 104‑0045 Japan; 11 Department of Breast Surgery, Graduate School of Medicine, Kyoto University, 54 Shogoin-Kawahara-cho, Sakyo-ku, Kyoto, 606-8507 Japan; 12 Department of Breast Surgery, National Hospital Organization Tokyo Medical Center, 2-5-1, Higashigaoka, Meguro-ku, Tokyo 152-8902 Japan; 13 Department of Surgical Oncology, Research Institute for Radiation Biology and Medicine, Hiroshima University, 1-2-3 Kasumi, Minami-Ku, Hiroshima City, Hiroshima 734-8551 Japan; 14 Department of Medical Oncology, Fukushima Medical University, Fukushima, Japan

**Keywords:** Body mass index, Breast cancer registry, Japanese Breast Cancer Society, National clinical database, Statistics, Subtype

## Abstract

The Japanese Breast Cancer Society initiated the breast cancer registry in 1975 and migrated the registry to the National Clinical Database-Breast Cancer Registry (NCD-BCR) in 2012. This annual report presents 2021 data on the NCD-BCR. We analyzed data from 98,540 breast cancer (BC) cases registered in 2021. In 2021, 99.4% of BC cases were females with a median age of 61. Most (57.5%) were diagnosed at early stages (Stage 0 or I). Breast-conserving surgery was performed in 42.8% of cases. Sentinel lymph node biopsy was performed in 67.8%, followed by radiotherapy in 71.0% of those post-conserving surgery. Regarding postoperative systemic therapy, 63.1% received endocrine therapy, 28.2% received chemotherapy, and 14.9% received molecular-targeted therapy. ER positivity was observed in 75.2%, HER2 in 13.6%, and Ki67 ≥30% in 29.1% of cases. The median age of premenopausal cases was 46 (interquartile range, 42–49) years and the median BMI was 21.5 (19.7–24.2) kg/m^2^ whereas the median age of postmenopausal cases was 69 (61–76) years and the median BMI was 23.0 (20.6–25.9) kg/m^2^. In premenopausal cases, cases with normal BMI were more likely to be found at checkups without subjective symptoms and in the early stage than those with high BMI. The tendency of ER, PgR, HER2, and Ki67 status on BMI differed by menopause status; premenopausal cases with a lower BMI showed higher proportions of ER- and PgR-positive cancer and lower proportions of cancer with high Ki67. These nationwide descriptive statistics would help clinical explanation and further research on breast cancer.

## Preface

The Japanese Breast Cancer Society (JBCS) initiated a breast cancer registry in 1975. In 2004, this registry transitioned to a web-based system in collaboration with a non-profit organization [1]. In 2012, the registry was migrated to the National Clinical Database-Breast Cancer Registry (NCD-BCR). Further details of the registry were outlined in previous annual reports [2–5].

Facilities across Japan have been registering cases diagnosed with new-onset breast cancer to the NCD-BCR since 2012. By utilizing NCD-BCR data, institutions can obtain certification for surgical specialty training programs, and surgeons can either acquire or renew their professional licenses [4]. The JBCS also developed quality indicators with the NCD-BCR office for feedback to treatment providers of breast cancer. Additionally, the registry allows various nationwide retrospective research [6–21]. For further use of this registry, its descriptive statistics are necessary.

Obesity is reported to be a risk of postmenopausal breast cancer [22, 23]. Regarding premenopausal breast cancer, obesity can be associated with a decreased risk [24, 25]; however, the association may be unclear in the Asian population [23]. Furthermore, although previous reports indicated that the association between body mass index (BMI) and the risk can differ by type of breast cancer [22, 24–26], little evidence is available.

This annual NCD-BCR report presents an overview of 98,540 breast cancer cases registered from 1,390 institutes in 2021. Additionally, we described characteristics of cases categorized by menopause status and BMI.

## Background characteristics

**Fig. 1:**
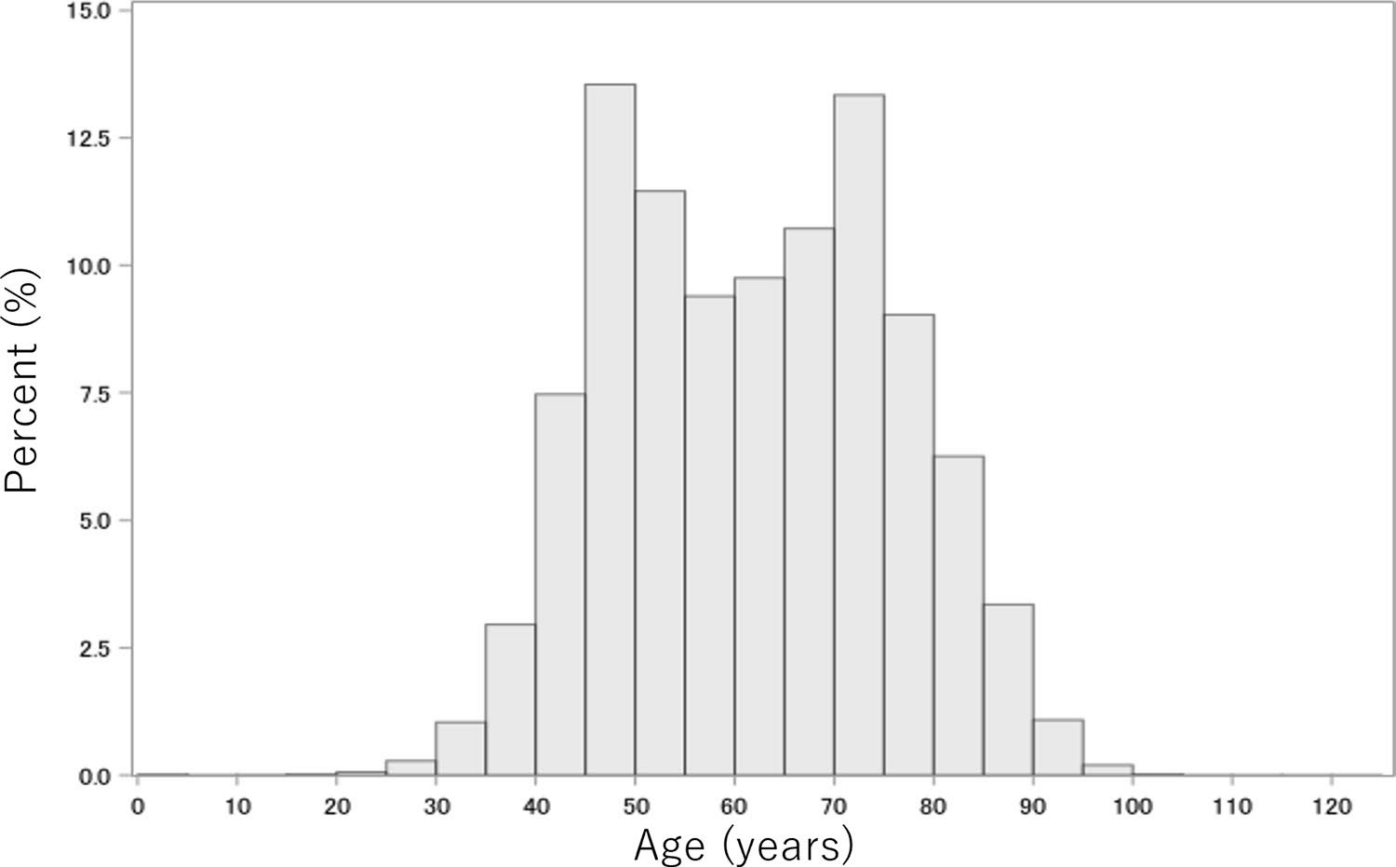
Distribution of age at the time of cancer diagnosis

Of 98,540 breast cancer cases, 97,960 (99.4%) were female. The median age at cancer diagnosis was 61 years with an interquartile range (IQR) from 49 to 73 years (Table 1); moreover, 44% (43,096 cases) were aged ≥ 65 (Fig. 1). The geographical distribution in Japan was as follows: 4.6% in Hokkaido, 6.2% in Tohoku, 35.7% in Kanto, 15.2% in Chubu, 5.6% in Chugoku, 2.9% in Shikoku, and 10.6% in Kyushu/Okinawa. Synchronous bilateral breast cancer was observed in 6,859 cases (7.0%), and a family history of breast cancer was noted in 18,111 cases (18.5%). Regarding clinical stage (cStage), 56,356 cases (57.5%) were diagnosed with early-stage breast cancer of Stage 0 or I.

**Table 1:** Background characteristics of 97,960 female breast cancer

Patient characteristics				Cancer characteristics		
Area				Clinical T status		
Hokkaido	4,506	(4.6)		Tis	14,746	(15.1)
Tohoku	6,082	(6.2)		T0	405	(0.4)
Kanto	34,945	(35.7)		T1	44,774	(45.7)
Chubu	14,920	(15.2)		T2	28,968	(29.6)
Kinki	17,659	(18.0)		T3	3,050	(3.1)
Chugoku	5,521	(5.6)		T4	4,589	(4.7)
Shikoku	2,879	(2.9)		Unknown	1,427	(1.5)
Kyushu	10,341	(10.6)		Clinical N status		
Age, years	61	(49–73)		N0	80,167	(81.8)
Bilateral				N1	12,373	(12.6)
Synchronous	6,859	(7.0)		N2	1,949	(2.0)
Metachronous	4,194	(4.3)		N3	2,050	(2.1)
Family history				Unknown	1,420	(1.4)
Presence	18,111	(18.5)		Clinical M status		
Absence	74,925	(76.5)		M0	94,342	(96.3)
Unknown	4,924	(5.0)		M1	1,729	(1.8)
Menstrual status				Unknown	1,888	(1.9)
Premenopausal	29,819	(30.4)		Clinical stage		
Postmenopausal	65,542	(66.9)		0	14,667	(15.0)
Unknown	2,597	(2.7)		I	41,689	(42.6)
BMI, kg/m^2^				IIA	23,016	(23.5)
<18.5	9,459	(9.7)		IIB	7,766	(7.9)
18.5–24.9	60,827	(62.1)		IIIA	2,282	(2.3)
25.0–29.9	20,521	(20.9)		IIIB	3,148	(3.2)
30.0–34.9	5,115	(5.2)		IIIC	1,571	(1.6)
35.0–39.9	1,047	(1.1)		IV	1,729	(1.8)
≥40.0	311	(0.3)		Unknown	2,092	(2.1)
Unknown	680	(0.7)				

Data are shown in n (%) or median (interquartile range). TNM classifications were identified using the UICC staging system

*BMI* body mass index

## Pathology

The pathological assessment of surgical specimens for 97,286 female cases (regardless of preoperative therapy) revealed that 11,357 (11.7%) were finally diagnosed as pTis. The most frequent range of invasive tumor size was from 1.1 to 2.0 cm (28,052 cases, 28.8%). Of 87,619 cases who received surgery on axillary lymph nodes, 76.8% had no axillary lymph node metastasis (pN0) whereas 6.1% had metastasis in ≥ 4 axillary nodes.

Among 97,960 female cases with breast surgery, 75.2% were estrogen receptor (ER) positive (i.e., ≥ 10% positive cells), 62.3% were progesterone receptor (PgR) positive, and 13.6% were Human Epidermal Growth Factor Receptor 2 (HER2) positive. Of 82,563 cases where Ki67 was measured, 29.1% exhibited a Ki67 positivity proportion ≥ 30%.

## Breast cancer treatments

Surgical procedures are described in Table 2. Among 94,342 female cases without distant metastasis, 40,384 (42.8%) underwent a breast-conserving mastectomy. A total of 70,997 cases (75.2%) underwent sentinel biopsy; subsequently, 7,014 (9.9%) had axillary node dissection among them. Breast reconstruction was performed in 5,996 cases (11.7% of 51,352 mastectomies). Among 1,540 cases of autologous reconstruction, a pedicled latissimus dorsi musculocutaneous flap was a dominant procedure.

**Table 2:** Surgical procedures for 94,342 female cases without distant metastasis

	n	(%)
Breast-conserving surgery	40,384	(42.8)
Mastectomy	47,067	(49.9)
Nipple sparing mastectomy	2,274	(2.4)
Skin sparing mastectomy	2,011	(2.1)
Others	415	(0.4)
Procedures for axillary lymph nodes		
Sentinel biopsy	63,983	(67.8)
Sentinel biopsy to dissection	7,014	(7.4)
Dissection without biopsy	13,750	(14.6)
Sampling	1,078	(1.1)
Reconstruction		
Tissue expander	3,838	(64.0)
Implant	299	(5.0)
Autologous	1,540	(25.7)
Pedicled RAMF	132	(2.2)
Free RAMF	509	(8.5)
Pedicled LDF	857	(14.3)
Free LDF	42	(0.7)
Others	319	(5.3)

*RAMF* rectus abdominis musculocutaneous flap, *LDF* latissimus dorsi musculocutaneous flap

^*^The percentage denominator is 5,996, the total number of reconstruction cases

Perioperative treatments for female cases without distant metastasis are outlined in Table 3. Whereas 40,384 cases underwent a breast-conserving mastectomy, 28,668 (71.0%) received whole breast irradiation; additionally, 2,236 (5.5%) received radiotherapy to both the whole breast and regional lymph nodes. Whereas 47,067 cases underwent a mastectomy, 649 (1.4%) received radiotherapy to the chest wall alone and 5,121 (10.9%) received radiotherapy to the chest wall and regional lymph nodes. Regarding systemic therapies, 22,258 (23.6%) received neoadjuvant chemotherapy. Additionally, 63.1% received postoperative endocrine therapy, 28.2% received adjuvant chemotherapy, and 14.9% received adjuvant molecular-targeted therapy.

**Table 3:** Perioperative treatments for 94,342 female cases without distant metastasis

	Preoperative		Postoperative	
	n	%	n	%
Radiotherapy	–	–	37,673	(39.9)
Whole breast alone	–		28,668	(30.4)
Whole breast + regional lymph nodes	–		2,236	(2.4)
Chest wall alone	–		649	(0.7)
Chest wall + regional lymph nodes	–		5,121	(5.4)
Regional lymph nodes alone	–		210	(0.2)
Others	–		789	(0.8)
Endocrine therapy	4,067	(4.3)	59,506	(63.1)
AIs	2,520	(2.7)	36,284	(38.5)
SERMs	1041	(1.1)	17,932	(19.0)
AIs/SERMs + LHRHa	188	(0.2)	4,265	(4.5)
Others	318	(0.3)	1025	(1.1)
Chemotherapy	22,258	(23.6)	26,637	(28.2)
Anthracyclines	10,315	(10.9)	8,886	(9.4)
Taxanes	10,874	(11.5)	9,384	(9.9)
Docetaxel + cyclophosphamide	313	(0.3)	4,527	(4.8)
Capecitabine	23	(0.0)	1,724	(1.8)
Tegafur/gimeracil/oteracil	48	(0.1)	693	(0.7)
Carboplatin	165	(0.2)	99	(0.1)
Others	520	(0.6)	1,324	(1.4)
Molecular targeted therapy	9,346	(9.9)	14,095	(14.9)
Trastuzumab	4,801	(5.1)	7,827	(8.3)
Pertuzumab	4,256	(4.5)	4,412	(4.7)
Trastuzumab emtansine	30	(0.0)	1253	(1.3)
Bevacizumab	152	(0.2)	41	(0.0)
Olaparib	1	(0.0)	20	(0.0)
Others	106	(0.1)	542	(0.6)

*AI* aromatase inhibitors, SERM selective estrogen receptor modulator, LHRHa luteinizing hormone-releasing hormone agonists

### Characteristics categorized by menopause status and body mass index

**Fig. 2:**
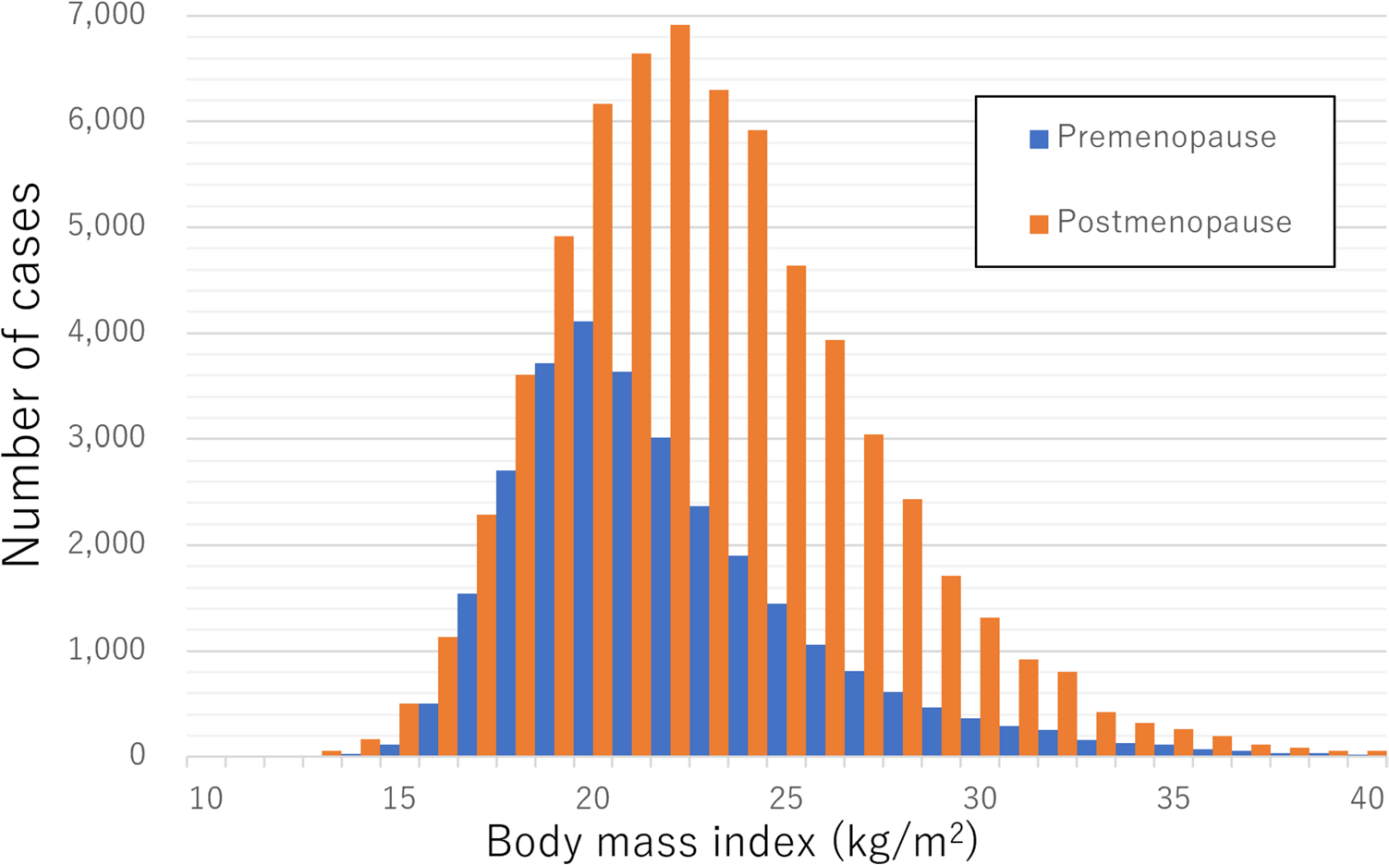
Distribution of body mass index at the time of cancer diagnosis categorized by menopause status

We identified female cases with menopausal status and categorized them by their BMI according to a previous study; cases with outliers of BMI were excluded [24]. The median age of 29,627 premenopausal cases was 46 (IQR, 42–49) years and the median BMI was 21.5 (19.7–24.2) kg/m^2^ whereas the median age of 65,046 postmenopausal cases was 69 (61–76) years and the median BMI was 23.0 (20.6–25.9) kg/m^2^. The distribution of BMI is depicted in Fig. 2.

Table 4 shows the way to detect breast cancer categorized by BMI and menopause status. Regarding premenopausal cases, approximately 40% of cases with normal BMI (18.5–24.9 kg/m^2^) were found in a breast cancer screening without subjective symptoms. In contrast, cases with a BMI above 30 kg/m^2^ were likely to have self-detected breast cancer. Postmenopausal cases were more likely to be detected other than by self-detection or screening compared with premenopausal cases (approximately 20% vs. 10%), presumably due to detection in the examination of comorbidities accompanied by advanced age.

**Table 4:** Way to detect breast cancer categorized by body mass index and menopause status

	Self-detection		Breast cancer screening				Others		Missing data		
			With subjective symptoms		Without subjective symptoms						
Body mass index, kg/m^2^	n	%	n	%	n	%	n	%	n	%	Total
Premenopausal cases											
<18.5	1,653	(48.9)	271	(8.0)	1,071	(31.7)	381	(11.3)	7	(0.2)	3,383
18.5–21.9	5,893	(45.4)	906	(7.0)	4,849	(37.3)	1,304	(10.0)	38	(0.3)	12,990
22.0–24.9	3,303	(45.1)	552	(7.5)	2,715	(37.1)	713	(9.7)	37	(0.5)	7,320
25.0–29.9	2,102	(48.4)	329	(7.6)	1,493	(34.4)	407	(9.4)	14	(0.3)	4,345
30.0–34.9	665	(55.4)	72	(6.0)	359	(29.9)	102	(8.5)	2	(0.2)	1,200
35.0–39.9	171	(56.3)	19	(6.3)	83	(27.3)	31	(10.2)	0	(0.0)	304
≥40.0	45	(52.9)	11	(12.9)	18	(21.2)	11	(12.9)	0	(0.0)	85
Postmenopausal cases											
<18.5	3,219	(55.8)	261	(4.5)	1,122	(19.4)	1,128	(19.5)	43	(0.7)	5,773
18.5–21.9	10,127	(51.4)	1,033	(5.2)	5,186	(26.3)	3,309	(16.8)	63	(0.3)	19,718
22.0–24.9	9,702	(50.5)	955	(5.0)	5,135	(26.7)	3,335	(17.4)	78	(0.4)	19,205
25.0–29.9	8,087	(51.6)	690	(4.4)	3,974	(25.3)	2,848	(18.2)	81	(0.5)	15,680
30.0–34.9	1,910	(50.6)	172	(4.6)	918	(24.3)	753	(20.0)	20	(0.5)	3,773
35.0–39.9	359	(50.8)	33	(4.7)	173	(24.5)	140	(19.8)	1	(0.1)	706
≥40.0	98	(51.3)	9	(4.7)	34	(17.8)	49	(25.7)	1	(0.5)	191

Regardless of menopause status, cases with a lower BMI were likely to have earlier stages of breast cancer (Table 5). Whereas approximately 60% of premenopausal cases with a low or normal BMI (< 30 kg/m^2^) had cancer with stage 0 or I, premenopausal cases with a BMI above 30 kg/m^2^ were likely to have cancer with stage II, III, or IV. This tendency may be associated with the way to detect as shown above.

**Table 5.** Clinical cancer stage categorized by body mass index and menopause status

Body mass index, kg/m^2^	Stage 0		Stage I		Stage II		Stage III		Stage IV		Missing data		Total
	n	%	n	%	n	%	n	%	n	%	n	%	
Premenopausal cases													
<18.5	764	(22.6)	1,503	(44.4)	795	(23.5)	176	(5.2)	50	(1.5)	95	(2.8)	3,383
18.5–21.9	2,754	(21.2)	5,739	(44.2)	3,502	(27.0)	579	(4.5)	118	(0.9)	298	(2.3)	12,990
22.0–24.9	1,331	(18.2)	3,022	(41.3)	2,254	(30.8)	462	(6.3)	100	(1.4)	151	(2.1)	7,320
25.0–29.9	667	(15.4)	1,613	(37.1)	1,574	(36.2)	319	(7.3)	71	(1.6)	101	(2.3)	4,345
30.0–34.9	175	(14.6)	392	(32.7)	477	(39.8)	110	(9.2)	25	(2.1)	21	(1.8)	1,200
35.0–39.9	55	(18.1)	85	(28.0)	126	(41.4)	30	(9.9)	6	(2.0)	2	(0.7)	304
≥40.0	4	(4.7)	37	(43.5)	29	(34.1)	11	(12.9)	3	(3.5)	1	(1.2)	85
Postmenopausal cases													
<18.5	787	(13.6)	2,400	(41.6)	1,699	(29.4)	581	(10.1)	177	(3.1)	129	(2.2)	5,773
18.5–21.9	2,699	(13.7)	8,824	(44.8)	5,889	(29.9)	1,556	(7.9)	397	(2.0)	353	(1.8)	19,718
22.0–24.9	2,468	(12.9)	8,352	(43.5)	6,331	(33.0)	1,427	(7.4)	309	(1.6)	318	(1.7)	19,205
25.0–29.9	1,943	(12.4)	6,521	(41.6)	5,495	(35.0)	1,169	(7.5)	260	(1.7)	292	(1.9)	15,680
30.0–34.9	425	(11.3)	1,543	(40.9)	1,377	(36.5)	284	(7.5)	74	(2.0)	70	(1.9)	3,773
35.0–39.9	69	(9.8)	295	(41.8)	260	(36.8)	57	(8.1)	19	(2.7)	6	(0.8)	706
≥40.0	22	(11.5)	65	(34.0)	82	(42.9)	17	(8.9)	3	(1.6)	2	(1.0)	191

**Fig. 3:**
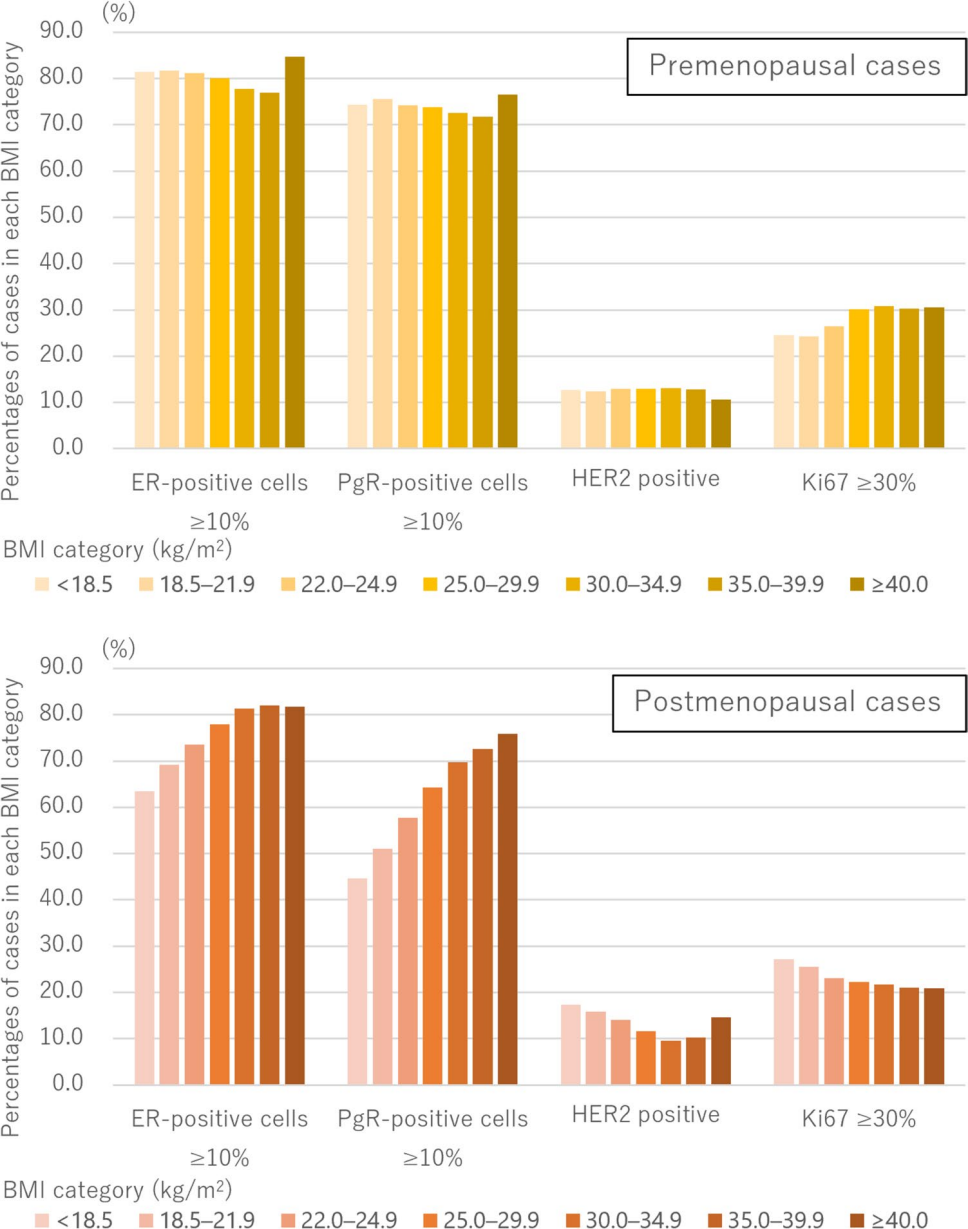
Pathological status categorized by body mass index and menopause status. *BMI* Body mass index, *ER* Estrogen receptor, *PgR* Progesterone receptor, *HER2* Human Epidermal growth factor receptor 2

Figure 3 depicts ER, PgR, HER2, and Ki67 status of breast cancer categorized by BMI and menopause status. Regarding premenopausal cases, cases with a lower BMI showed higher proportions of ER- and PgR-positive cancer. The results are plausible because a low BMI would be a risk of premenopausal luminal-type breast cancer [22, 24–26]. Furthermore, cases with a lower BMI were less likely to have cancer with high Ki67. In postmenopausal cases, conversely, cases with a lower BMI were less likely to have ER- and PgR-positive cancer and cancer with low Ki67; moreover, they were more likely to have HER2-positive breast cancer compared with cases with a normal BMI or premenopausal status.

## Postscript

The detailed data regarding breast cancer, available within the Japanese Breast Cancer Registry, is closely based on voluntary contributions by healthcare professionals, including physicians, across Japan. We sincerely acknowledge and appreciate their dedication in inputting this information. Their collective efforts allow for analyzing large-scale data, conducting further research, and ultimately improving the lives of patients with breast cancer. We anticipate that the current annual nationwide report will support physicians and scientists in comprehending and exploring breast cancer.

## Data Availability

The National Clinical Database-Breast Cancer Registry is not publicly accessible owing to the sensitive nature of the clinical information. Access was granted to a designated statistician through an application process approved by the relevant academic societies. Therefore, we are unable to share the original data from the present study.
